# Looking Beyond Distress: A Call for Spanning the Continuum of Mental Health Care

**DOI:** 10.7759/cureus.2815

**Published:** 2018-06-15

**Authors:** Seema Mehrotra, Neha Swami

**Affiliations:** 1 Clinical Psychology, National Institute of Mental Health and Neurosciences, Bangalore, IND; 2 Clinical Psychology, National Institute of Mental Health and Neurosciences , Bangalore , IND

**Keywords:** well-being, distress, mental health promotion, emotional well-being, psychological well-being, social well-being

## Abstract

Background

Despite mental health’s definition as a state of well-being, mental health research and practice has often narrowly focused on pathology and distress. The identification, treatment, and rehabilitation of mental illness are the primary activities pertaining to the role of mental health professionals, but only focusing on these primary activities carries the risk of neglecting other legitimate responsibilities related to prevention of illness and promotion of wellness. Therefore, it becomes important to highlight the potential utility of separately examining levels of distress, alongside levels of well-being in community samples. The goal of this study was to determine the extent to which low levels of distress or a lack of significant distress co-occurs with high levels of well-being in a sample of urban Indian adults.

Methods

Standardized measures of psychological distress, along with measures of emotional, psychological, and social well-being, were used in a sample of 300 adults aged 20 to 60 years. The participation of community-dwelling adults who could respond to the questionnaires in English or Hindi (the most widely spoken and official language in India) was solicited through posting announcements on various platforms in a metropolitan city in Southern India. The participants had the option to respond to the questionnaires via a paper-pencil version or through an online Google survey. Psychological distress was assessed using the Kessler Psychological Distress scale (K-10) on which a score over 19 indicated significant distress. We used the 25th and 75th percentile points on the respective distributions of scores to determine high, low, and moderate ranges on the three measures of well-being.

Results

Both men and women were well represented in the sample (47% and 53%, respectively). Approximately 41% were young adults, nearly 30% were in the 36 to 45-year age group, and 30% were in the 46 to 60-year age group. Approximately 48% of the overall sample (N = 143) exhibited an absence of significant distress. Patterns of well-being were examined in this group of low-distress participants. Only 13% of low-distress participants exhibited high emotional well-being (i.e., had a high positivity ratio) and high psychosocial functioning (i.e., had high psychological and social well-being). As many as 37.8% exhibited low well-being on at least one of the three measures of well-being, despite being low on distress. In other words, low well-being scores were fairly common in conjunction with non-significant levels of distress.

Conclusion

Reliance on low distress for operationalization of mental health may mask lower levels of emotional well-being and psychosocial functioning in a given population. The findings underscore the need to span the entire continuum of mental health care by routinely including preventive and promotive aspects in research and practice.

## Introduction

Mental health is defined as “a state of well-being in which the individual realizes his or her own abilities, can cope with the normal stresses of life, can work productively and fruitfully, and is able to make a contribution to his or her community” [1]. This definition of mental health comes close to how well-being is defined in the field of psychology in terms of feeling (i.e., emotional well-being) and functioning (i.e., psychosocial functioning). Emotional well-being (i.e., low frequency of negative emotions and high frequency of positive emotions), along with cognitive evaluations of life as satisfying, is also known as subjective well-being—the most examined form of well-being. There is voluminous empirical research on determinants and outcomes of emotional well-being [2]. Positive and negative emotions are independent constructs rather than opposites, and they have somewhat independent correlates [3]. The absence of negative emotions does not imply the presence of positive emotions and vice versa. Similarly, amelioration of distress does not necessarily enhance the experience of positive emotions [4]. These observations highlight the need to examine the balance of positive and negative emotions as reflected in a positivity ratio. Notwithstanding the controversies surrounding what may be the optimal positivity ratio, a large amount of research across divergent domains (e.g., couple relationships and team-level and individual functioning) indicates beneficial outcomes are associated with a ratio tilted in favor of positive over negative emotions [5-6]. Psychosocial functioning is examined in terms of psychological and social well-being. Psychological well-being itself is theorized as a multidimensional construct, with Ryff’s model being the most popular. In this model, psychological well-being is composed of six dimensions: self-acceptance, positive relations with others, personal growth, purpose in life, environmental mastery, and autonomy [7]. Well-being also needs to be examined beyond the personal sphere to incorporate high functioning with respect to social criteria. This idea is encompassed in the construct of social well-being. Individuals high in social well-being are those who see society as meaningful and possessing a growth potential, feel a belonging to and acceptance in their communities, and see themselves as contributing to society in some way [8].

Despite the broad definition of mental health as proposed by the World Health Organization and studies highlighting the significant benefits associated with high levels of well-being, research and practice in the field of mental health maintain a narrower focus on pathology and distress [9]. Examining levels of well-being, alongside levels of distress in community samples, is important and can highlight what we may be missing when we rely exclusively on distress levels to identify mental health needs in a given population. The goal of this study was to examine the extent to which low distress co-occurs with high levels of well-being in a community sample of urban Indian adults.

## Materials and methods

Sampling

The present study is part of ongoing research on the development of an internet-based tool for assessing well-being and is limited to examining the co-occurrence of low distress with high well-being. The study was initiated after obtaining approval from the National Institute of Mental Health and Neurosciences Institute Ethics Committee. The study was conducted in a metropolitan city in South India. We solicited the participation of adults aged 20 to 60 years old who possessed at least a high school education and were conversant in English or Hindi (the most widely spoken and official language in India) through the posting of announcements on various platforms (e.g., websites, mailers, and posters in institutes of higher education and workplaces). Those who provided informed consent were enrolled in the study. Hindi versions of the study measures were developed using standard translation and back-translation procedures. The participants had an option to respond to standardized questionnaires on distress and well-being via paper-pencil hard copy or via online Google survey.

Measures

We developed a basic data sheet to obtain sociodemographic information. We also assessed whether participants felt they were suffering from a mental health problem and if they were currently seeking professional help for a mental health problem. Psychological distress was assessed using the Kessler Psychological Distress Scale (K-10) composed of 10 items [10]. A cut-off score of over 19, as suggested by Andrew and Slade [11], was used for identifying participants in distress. Participants scoring 10 to 19 were considered well, a score of 20 to 24 indicated mild distress, a score of 25 to 29 indicated moderate distress, and a score of 30 to 50 indicated severe distress [10]. The 26-item revised Positive and Negative Affect Schedule (PANAS) [12-13] was used to obtain positive and negative affect scores and calculate the positivity ratio. The positivity ratio was calculated using the guidelines suggested by Fredrickson and Losada [14]. Positive and negative affect and the positivity ratio were used as indicators of emotional well-being. Psychological well-being was assessed using a measure adapted and standardized for use in Indian samples [15]; it consists of 20 items and four factor-based subscales: a sense of mastery and competence, positive relations, self-acceptance, and a sense of engagement and growth. Only the total psychological well-being scores were used in our analyses for the sake of parsimony. Social well-being was assessed using a measure consisting of nine items that capture a sense of social acceptance, social integration, social contribution, and social integration. Each item is a pair of binary statements, placed at opposite ends of a six-point response continuum depicted as ‘a’ to ‘f’ (e.g., ‘think I am doing a lot of things for the good of others/for society’ vs. ‘Right now I do not think I am able to focus beyond myself’). The respondent chooses a response depending on the extent to which his/her view is closer to any one of the two extreme/end-statements. This measure was developed as part of an Indian study wherein its internal consistency reliability and convergent validity (in terms of association with measures of youth engagement, web of connections and self-reported frequency of informal help-giving) were satisfactory (Michael RJ: Social well-being in Indian youth--unpublished doctoral thesis, National Institute of Mental Health and Neurosciences. Bangalore, India, 2015). Also, in the present study, this nine-item measure had satisfactory internal consistency with an alpha of 0.79 and social well-being scores were significantly correlated with measures of positive affect (correlation coefficient (r) = 0.28, p < 0.01) and psychological well-being (r = 0.44, p < 0.01). 

All the data analysis were carried out using the Statistical Package for Social Sciences (SPSS), version 16.0. Descriptive statistics were run to obtain the frequencies, percentages, and percentile points. Normality of score-distributions was examined on Kolmogorov Smirnov Test. Reliabilities of the measures have been determined in terms of internal consistency and reported as alpha values.

## Results

Sample characteristics

Both men and women were well represented in the overall sample. Approximately 41% were young adults, 28% were in the 36 to 45-year-old group, and 29% were in the 46 to 60-year old group. Both the genders were represented fairly in each age subgroup. Fifty-nine percent were married, and 51% were college graduates. Nineteen percent reported a monthly income of at least 25,000 Indian rupees, while 21% reported a monthly income of 65,000 Indian rupees or more. Another 20% indicated they were not earning an individual income. Sixty-seven percent of the participants were working, and 18% were student youths. Seven percent reported suffering from a mental health problem, while 12% reported being unsure if they had a mental health problem. Only 2% of responders reported consulting a mental health professional (Table 1).

**Table 1:** Basic Sample Characteristics (N = 300) N: number; SD: standard deviation; PUC: pre-university course.

		N = 300	Percent 100%
Gender	Male	140	46.7
	Female	158	52.7
Age Mean (SD)	20 - 35 years	124	41.33
	36 - 45 years	85	28.33
	46 - 60 years	87	29
Education	PUC	12	4
	Graduation/Diploma	153	51
	Post-graduation	132	44
Religion	Hindu	218	72.7
	Muslim	19	6.3
	Christian	54	18.0
	Sikh	3	1.0
	Others	3	1.0
Marital Status	Single	106	35.3
	Married	176	58.7
	Divorced/Separated	11	3.7
Work Status	Student	54	18.0
	Employed/Business	200	66.7
	Homemaker	20	6.7
	Retired	4	1.3
	Looking for paid job	4	1.3
Monthly Income (rupees)	Below 15,000	29	9.7
	15,000 - 25,000	28	9.3
	25,001 - 45,000	55	18.3
	45,001 - 65,000	21	7.0
	More than 65,000	62	20.7
	Not applicable	64	21.3
Suffering from some kind of mental health problem	Yes	20	6.7
	No	236	78.7
	Not sure	37	12.3
Currently consulting a mental health professional	Yes	5	1.7
	No	286	95.3

Distress and well-being measures

We used multiple measures of well-being to examine the extent to which low distress co-occurs with high levels of well-being. Scores obtained on all the measures had a fair spread across the possible range of scores, and the total scale reliabilities were 0.79 or higher. On K-10, the average score of the sample was near the mild distress cut-off (i.e., 20). Psychological well-being subscales also had satisfactory reliabilities, with alpha coefficients ranging from 0.69 to 0.84. Scores on negative affect, positivity ratio, social well-being, psychological distress, and two subscales of psychological well-being were non-normally distributed (Table 2).

**Table 2:** Descriptive Statistics on Study Measures (N = 300) *p < 0.05; **p < 0.01. K-10: Kessler Psychological Distress Scale; KS-Z: Kolmogorov-Smirnov Z value; NA: not applicable.

Variable	Min-Max possible	Min-Max obtained	Mean	Standard deviation		KS-Z	Alpha
Positive affect	13 - 65	13 - 65	41.45	10.22		0.87	0.90
Negative Affect	13 - 65	13 - 64	27.66	8.97		1.54*	0.86
Positivity Ratio	NA	0-13	1.77	2.06		4.23**	NA
Psychological Well-being Total	20 - 120	40 - 120	87.82	16.69	0.85		0.87
Social Well-being	9 - 54	14 - 54	39.92	7.69	1.66**		0.79
Psychological Distress (K-10)	10 - 50	10 - 49	20.73	7.39	1.61*		0.89

Distress levels in the overall sample

Forty-eight percent of the sample reported no significant distress, 25% reported mild distress, and 28% reported moderate to severe distress (Table 3). Nearly half the sample (48.3%) could be classified as experiencing low distress.

**Table 3:** Spread of Scores on Psychological Distress (N = 296*) *Data missing from 4 of 300 participants

Severity of distress	Frequency	Percentage
None/minimal	143	48.30
Mild	70	23.65
Moderate	41	13.85
Severe	42	14.19

Levels of well-being 

Scores in the high, low, and moderate range on the three measures of well-being were determined using the 25th and 75th percentile points on the respective distributions (Table 4). “Low well-being” refers to any score below the 25th percentile on the respective well-being measures. “High well-being” refers to any score above the 75th percentile.

**Table 4:** Percentage of Participants (N = 300) at Various Levels of Well-being Across Different Measures* *Based on percentile cut-offs

Levels of well-being	Score range and frequency	Positivity Ratio	Psychological Well-being	Social Well-being
Low	Score range	≤ 0.75	≤ 75	≤ 36
	Frequency (%)	75 (25%)	78 (26%)	79 (26.3%)
Moderate	Score range	0.75 - 1.86	75 - 101	36 - 45
	Frequency (%)	156 (52%)	144 (48%)	143 (47.7%)
High	Score range	≥ 1.86	≥ 101.0	≥ 45
	Frequency (%)	69 (23%)	78 (26%)	78 (26%)

The group of participants scoring 19 or below on the K-10 scale (i.e., the low-distress group) formed the basis for all subsequent analyses. As depicted in Table 5, the presence of high well-being (on any of the three measures) was evident in 35% to 42% of the low-distress participants. Low scores on psychological (9.8%) and social well-being parameters (16.1%) were more common than a low positivity ratio (7.7%) in the low-distress participants.

**Table 5:** Levels of Well-being in Low Distress Participants (N = 143)

Frequency			
Well-being Measures	Low	Moderate	High
Positivity Ratio	11 (7.7%)	77 (54%)	55 (38.5%)
Psychological Well-being	14 (9.80%)	69 (48%)	60 (42%)
Social Well-being	23 (16.1%)	70 (49%)	50 (35%)

Pattern of co-occurrence of low distress and low well-being

We cross-tabulated well-being levels in the low-distress individuals and found that 37.8% (54 of 143) exhibited low well-being on at least one of the three measures of well-being, despite being low on distress. This included 25.2% of the participants who were low on one of the three measures of well-being, 10.5% who were low on any two of the three well-being measures, and 2.1% who were low on all measures.

Pattern of co-occurrence of low distress with high well-being

Ninety-seven of 143 (67.8%) low-distress participants had high levels of well-being on at least one of the three well-being measures. In only 19 of the 143 (13.3%) participants, low distress co-occurred with high levels of psychological and social well-being as well as a high positivity ratio. While 38 participants (26.6%) exhibited a combination of high positivity ratio and high psychological well-being, 26 (18.2%) had high psychological and social well-being, and 23 (16.1%) exhibited a combination of a high positivity ratio, along with high social well-being. Seventeen percent of these low-distress participants had well-being scores in the moderate range (i.e., between the 25th and 75th percentiles) on all three measures.

## Discussion

We examined distress and well-being in a comprehensive manner in a sample of urban Indian adults. Keyes proposed the conceptualization and measurement of mental health should include not merely the absence of mental illness but the presence of positive mental health features, including emotional, psychological, and social well-being [16]. In a landmark study of midlife in adults in the United States (MIDUS), about 18% of participants fit the criteria for flourishing, which is broadly understood as involving high emotional, psychological, and social well-being. When cross-tabulated with the absence of mental illness, 16.6% of the study population had complete mental health. Complete mental health was predictive of several beneficial outcomes, such as fewer limitations of activities of daily living, fewer missed days of work, clear life goals, higher levels of resilience, and intimacy [17]. Looking beyond distress can widen the sphere of action to include mental health endeavors of relevance to a larger segment of the population.

Of the participants in our study with low levels of distress, only about 13% exhibited high emotional well-being (i.e., had a high positivity ratio) and high psychosocial functioning (i.e., had high psychological and social well-being). This figure is somewhat close to the prevalence of flourishing reported in the MIDUS study [17]; the difference observed is not surprising, given the differences in terms of various measurements used and the operationalization of high scores [18].

Low well-being scores in our study were often reported in conjunction with non-significant levels of distress. Thirty-eight percent of people in the low-distress group exhibited low well-being on at least one of the three measures, and 10% exhibited low well-being on two of the three well-being measures.

Our results support looking beyond distress scores to arrive at a comprehensive understanding of mental health in terms of emotional and psychosocial functioning. High positivity ratios signify not just lower levels of negative emotions but also higher levels of positive emotions. The frequent experience of positive emotions over time is theoretically linked with a broadening of attention and behavior repertoires and building of physiological, psychological, and social resources which may lead to a variety of positive outcomes. This line of theorizing is supported in several studies examining health and functioning outcomes associated with high positivity [6, 14, 19].

In this study, psychological well-being was examined as a whole composed of four components: a sense of mastery and competence, self-acceptance, positive relations, and a sense of engagement and growth. These components may serve as internal resources for individuals to recover from and cope with challenging life situations and exhibit resilient outcomes [20]. This suggests high scores on psychological well-being assessments may signal not only high psychological functioning but also the presence of protective factors for mental health. Similarly, high scores on social well-being assessments are likely to reflect a high sense of social connectedness and acceptance that can boost social resources and act as a buffer against stressful situations [21].

Owing to a dominant focus on pathology and distress in research and practice [9], mental health professionals are likely to see the identification, treatment, and rehabilitation of mental illness as their primary duties. Although these functions are crucial for mental health professionals, an exclusive focus on these comes with the risk of neglecting their other legitimate functions. There is a steadily growing body of evidence on the utility of mental health promotion and preventive measures [9, 22-23] with a call to broaden research attention beyond risk factors to include protective and promotive factors and to explore the public health benefits of this broadened approach [24]. Patel and Goodman made a strong case for research on both the individual and group level of protective factors and promotive interventions [25].

Our findings further support the need to address the continuum of mental health care in the field of research and practice. Reliance on low distress for operationalization of mental health may mask lower levels of emotional well-being and psychosocial functioning in a given population. Interventions that target improving a positivity ratio and psychosocial well-being may as serve preventive and promotive functions for mental health. The potential benefits of these interventions merit systematic research attention.

Focusing on promotive and preventive work may seem daunting—it implies a thinning of available professional resources and funding in already resource-scarce settings. In the long term, this apprehension may not be warranted. Efforts to reach the distressed population (with a probable diagnosis) not seeking professional help and the non-distressed population experiencing lower levels of well-being can overlap significantly [9]. Interventions aimed at enhancing one or more components of well-being can be of relevance to those experiencing distress [26]. Moreover, exposure to well-being interventions can also serve as a gateway to seek mainstream mental health services, thereby contributing to the goal of reducing the treatment gap, as participating in preventive and promotive interventions is likely to be less stigmatizing [27].

Psychiatry is the “branch of medicine focused on the diagnosis, treatment, and prevention of mental, emotional, and behavioral disorders” [28]. Similarly, clinical psychology “involves research, teaching, and services relevant to the applications of principles, methods, and procedures for understanding, predicting, preventing and/or alleviating intellectual, emotional, biological, psychological, social and behavioral maladjustment, disability, and discomfort applied to a wide range of client populations” [29].

Psychologists engage in primary, secondary, and tertiary interventions and work not just with persons having a mental illness but also with those without mental illness to promote adaptation, adjustments, and personal development [29]. Unfortunately, the emphasis on training and practice in mental health tends to adopt a narrower focus on understanding, identifying, and treating psychopathology in the clinical and community settings [9]. Sufficient and consistent attention to promotive and preventive aspects of mental health in training, practice, and research can bridge the gap between the definition of mental health and the activities undertaken by mental health professionals. This can result in cumulative gains in practical knowledge and help realize the potential of a strategy that entails spanning the entire continuum of mental health care.

The study has a few limitations. Emotional well-being was measured as the ratio of positive and negative affect; however, life satisfaction was not assessed. Scores on psychological and social well-being were analyzed in terms of totals. Future research should fine-tune the operationalization of mental health and flourishing. Larger studies with greater sample sizes representative of a given population are warranted. The study was a pilot exercise to determine the need for comprehensive assessments of mental health and was not intended to serve as providing estimates on the prevalence of well-being in urban Indian adults.

Notwithstanding the limitations, this is one of the first Indian studies to attempt to examine levels of well-being using multiple components in a sample of individuals with no significant distress. Our assessment of mental health was broadly in line with Keyes’ conceptualization of mental health as it used a combination of indices of distress, emotional well-being, and psychological and social well-being [17].

## Conclusions

Relying on a lack of significant distress as an indicator of mental health and well-being has inherent limitations. Mental health professionals need to widen their approach to mental health research and practice to routinely include preventive and promotive aspects—this would not only better align with the legitimate roles of mental health professionals but also help address the unmet mental health needs of a population.
